# Production of Satratoxin G and H Is Tightly Linked to Sporulation in *Stachybotrys chartarum*

**DOI:** 10.3390/toxins14080515

**Published:** 2022-07-28

**Authors:** Katharina Tribelhorn, Magdalena Twarużek, Ewelina Soszczyńska, Jörg Rau, Christiane Baschien, Reinhard K. Straubinger, Frank Ebel, Sebastian Ulrich

**Affiliations:** 1 Chair of Bacteriology and Mycology, Department of Veterinary Sciences, Faculty of Veterinary Medicine, Institute for Infectious Diseases and Zoonosis, LMU Munich, 80539 Munich, Germany; katharina.tribelhorn@micro.vetmed.uni-muenchen.de (K.T.); r.straubinger@lmu.de (R.K.S.); frank.ebel@micro.vetmed.uni-muenchen.de (F.E.); 2 Department of Physiology and Toxicology, Faculty of Biological Sciences, Kazimierz Wielki University, 85–064 Bydgoszcz, Poland; twarmag@ukw.edu.pl (M.T.); eweso@ukw.edu.pl (E.S.); 3 Chemical and Veterinary Analysis Agency Stuttgart, 70736 Fellbach, Germany; joerg.rau@cvuas.bwl.de; 4 Leibniz-Institute DSMZ-German Collection of Microorganisms and Cell Cultures, 38124 Braunschweig, Germany; christiane.baschien@dsmz.de

**Keywords:** *Stachybotrys chartarum* genotype S, sporulation, satratoxins, macrocyclic trichothecenes, inter-colony communication

## Abstract

*Stachybotrys chartarum* is a toxigenic fungus that is frequently isolated from damp building materials or improperly stored forage. Macrocyclic trichothecenes and in particular satratoxins are the most potent mycotoxins known to be produced by this fungus. Exposure of humans or animals to these secondary metabolites can be associated with severe health problems. To assess the pathogenic potential of *S. chartarum* isolates, it is essential to cultivate them under conditions that reliably promote toxin production. Potato dextrose agar (PDA) was reported to be the optimal nutrition medium for satratoxin production. In this study, the growth of *S. chartarum* genotype S strains on PDA from two manufacturers led to divergent results, namely, well-grown and sporulating cultures with high satratoxin concentrations (20.8 ± 0.4 µg/cm^2^) versus cultures with sparse sporulation and low satratoxin production (0.3 ± 0.1 µg/cm^2^). This finding is important for any attempt to identify toxigenic *S. chartarum* isolates. Further experiments performed with the two media provided strong evidence for a link between satratoxin production and sporulation. A comparison of three-point and one-point cultures grown on the two types of PDA, furthermore, demonstrated an inter-colony communication that influences both sporulation and mycotoxin production of *S. chartarum* genotype S strains.

## 1. Introduction

*Stachybotrys chartarum* occurs ubiquitously in the environment and is frequently found on dead plants (e.g., straw), other cellulosic (e.g., culinary herbs), and building materials (e.g., gypsum and wallpaper) [1,2,3]. The species *S. chartarum* can be subdivided into two distinct chemotypes based on the production of either atranones (chemotype A) or macrocyclic trichothecenes (MT; chemotype S) [4]. Alternatively, the three genotypes S, A, and H can be differentiated according to the presence or absence of genes that are presumed to encode relevant enzymes for the biosynthesis of these mycotoxins (*atr*1-14 and *sat*1-21) [4,5,6,7,8]. *S. chartarum* genotype S strains harbor the three satratoxin gene clusters SC1, SC2, and SC3 and have been implicated in several types of diseases [9,10,11,12,13,14,15]. In animals, stachybotryotoxicosis can occur after oral uptake of mycotoxins, especially in horses and less frequently in ruminants [16,17,18]. Humans, in particular infants, are primarily at risk after exposure to airborne toxins in water-damaged buildings [9,19]. Exposure to MT may cause pulmonary hemorrhage in infants or symptoms related to the sick building syndrome complex [9,20,21]. MT represents the most cell-toxic trichothecenes currently known and includes roridin E and L-2, verrucarin J, and satratoxin F, G, and H [22]. By binding irreversibly to the 60S ribosomal subunit, satratoxins inhibit protein biosynthesis and induce apoptosis in neuronal cell lines [23,24,25,26].

Despite the medical relevance of the MT of *S. chartarum*, little is known about the factors that influence their production and that are therefore of crucial importance for efficient diagnostics. Reliable protocols are needed to determine the ability of individual isolates to produce MT and thereby evaluate their hazard potential. Apart from cellulose-containing media, potato dextrose agar (PDA) was shown to be an excellent medium for MT production [27], but the nutritional factors that govern MT production have not been identified yet. Temperature and humidity have been shown to influence MT production, but the underlying mechanisms and regulatory genes are still unknown [6,28,29].

In other fungi, mycotoxin production and sporulation are often functionally linked. In *Aspergillus* spp. and *Fusarium* spp., both processes are regulated by similar G-protein signaling pathways [28,29]. These pathways commonly regulate fungal development, stress response, and the expression of virulence traits [30]. Sporulation can be influenced by many external factors, including molecules that are produced by other fungi, such as oxylipids or volatile organic components (VOC) [31,32]. For *S. chartarum*, all this is still uncharted territory, but research in this field is clearly necessary given the critical health hazard that especially satratoxins G, H, and F of all the above-mentioned MTs pose to humans and animals.

In this study, we have investigated the production of satratoxins G and H by three genotype S strains of *S. chartarum* on two commercially available PDA media; this resulted in two completely different phenotypes, one with high sporulation and strong satratoxin production and another almost without sporulation and satratoxin production. These data indicate that sporulation and satratoxin production are linked in *S. chartarum.* We, furthermore, observed that the presence of neighboring colonies of the same *S. chartarum* strain stimulated both sporulation and satratoxin production.

## 2. Results

**Figure 1:**
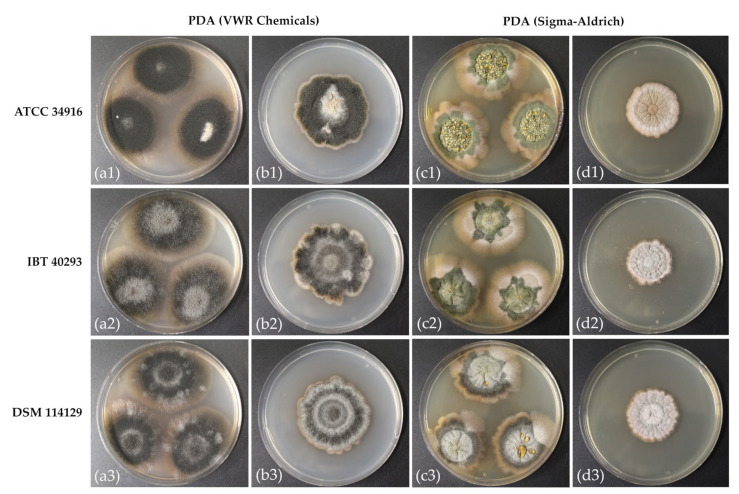
Colonies of *S. chartarum* genotype S strains ATCC 34916 (**a1**–**d1**), IBT 40293 (**a2**–**d2**), and DSM 114129 (**a3**–**d3**) were grown as three-point and one-point cultures on PDA-VWR (**a1**–**a3**) and (**b1**–**b3**) and PDA-Sigma (**c1**–**c3**) and (**d1**–**d3**). The images are representative of three parallel cultures for each condition.

The starting point of this study was the unexpected observation that the growth of *S. chartarum* genotype S strains on PDA of different manufacturers (VWR Chemicals and Sigma-Aldrich) resulted in colonies with surprisingly different phenotypic appearances (Figure 1). We compared both media using three *S. chartarum* strains that are known for their effective MT production [2,4,27]. These strains originate from three different habitats, namely building material (IBT 40293), animal feed (ATCC 34916), and food (DSM 114129). They were cultivated as three-point and one-point cultures on PDA with three technical replicates for 21 days in the dark with an a_w_ of 0.98 (each culture in triplicates *n* = 36 agar plates).

### 2.1. Colonies Formed under the Different Experimental Conditions

Although nominally identical, the two media differed in one component: VWR Chemicals (PDA-V) comprises potato peptone (4.0 g/L), whereas PDA-S from Sigma-Aldrich contains potato infusion (4.0 g/L). This difference is most likely responsible for the marked differences in growth and sporulation shown in Figure 1.

PDA was previously described as the optimum medium for mycotoxin production and sporulation of *S. chartarum* [1,27,33]. With respect to sporulation, this was true for PDA-V and three-point cultures, whereas PDA-S supported only limited sporulation (Figure 1). The difference was even more pronounced for one-point cultures: colonies on PDA-V sporulated although less well than those of the three-point cultures, whereas single colonies of all three strains showed a nearly complete lack of sporulation on PDA-S (Figure 1).

We next investigated whether sporulation and satratoxin production are linked in *S. chartarum.* We measured the colony size and determined the number of spores per cm^2^ colony area. In addition, we cultured the strains also in potato dextrose broth (VWR Chemicals), since *Stachybotrys* spp. is known to produce no spores in broth culture [34].

The colony areas of the three- and the one-point cultures grown on PDA-V were significantly larger than those of the corresponding colonies on PDA-S. This demonstrates that PDA-V supports better growth than PDA-S (Table 1). Interestingly, the single colonies on PDA-V were larger than the corresponding colonies of the three-point cultures (average difference of 2.0 cm^2^) most likely due to competition between the colonies (Table 1). Surprisingly, we observed an opposite effect on PDA-S: the colonies of the three-point cultures were 4.0 cm^2^ larger than the colonies of the corresponding one-point culture (Table 1). A microscopic comparison of the mycelia of these colonies revealed a much denser hyphal network on PDA-V, regardless of the number of colonies present on the agar plate (Figure S1). To rule out that the observed differences between the two PDAs were due to batch differences, we tested a second batch for each medium and obtained very similar results as in the initial experiments (data not shown).

**Figure 2:**
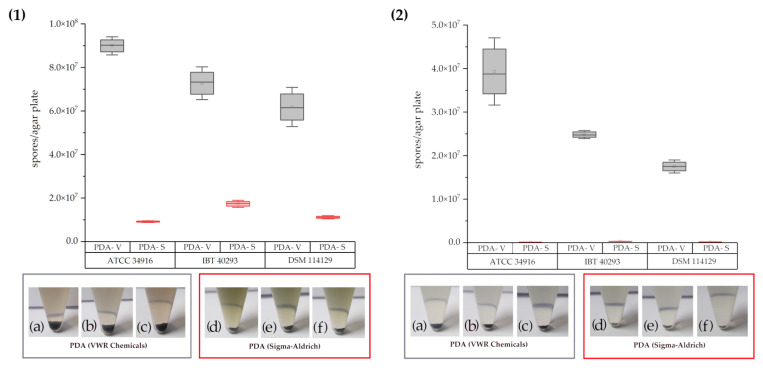
The spore count and images of conidial pellets of *S. chartarum* genotype S strains ATCC 34916 (**1(a)**/**1(d)** and **2(a)/2(d)**), IBT 40293 (**1(b)**/**1(e)** and **2(b)**/**2(e)**), and DSM 114129 (**1(c)**/**1(f)** and **2(c)**/**(f)**) obtained after growth on PDA-V (gray box plots) and PDA-S (red box plots). (1): three-point cultures, (2): one-point cultures. The spores were isolated from the cultures shown in Figure 1.

Apart from their size, the colonies also clearly differed in their level of sporulation. We, therefore, isolated the spores, counted them, and calculated the number of conidia per plate (Figure 2) and per cm^2^ colony area (Figure S2).

Not surprisingly, we found more spores per plate for three-point than for one-point cultures regardless of the PDA used. However, the picture becomes more complicated if the data are presented as spores per cm^2^ colony area. For two strains, IBT 40293 and DSM 114129, the colonies of the one-point cultures formed 20% and 18% fewer spores per cm^2^, respectively, than the colonies of the three-point cultures. However, strain ATCC 34916 behaved differently and produced about 30% more spores per cm^2^ in the one-point culture than in the three-point culture. These findings align well with the colony images shown in Figure 1.

All strains produced fewer spores on PDA-S than on PDA-V (Figure 1, Table S2). The PDA-S cultures generated consistently more spores per cm^2^ in the three-point cultures than in the one-point cultures. As shown in Figure S2, one-point cultures on PDA-S produced 92.5% fewer spores per cm^2^ than the corresponding three-point cultures and were, in fact, more or less devoid of spores. Figure 2 shows the pellets of the harvested conidia from the different plates. The spores formed a black pellet, and the size of these pellets correlates well to the number of spores obtained by counting. Since the number of colonies per plate was the only parameter that differed between the three- and the one-point cultures, our data demonstrate that the level of sporulation is not only determined by the medium but also by interactions between different colonies present on the same plate.

We also observed that three-point cultures on PDA-S produced a green pigment, whereas no such pigment was detectable for colonies on PDA-V. After centrifugation of the spores, the supernatant of the one-point cultures was almost clear, whereas brownish (Figure 1a–c and Figure 2) and greenish (Figure 1d–f and Figure 2) solutions were obtained from the three-point cultures.

**Figure 3:**
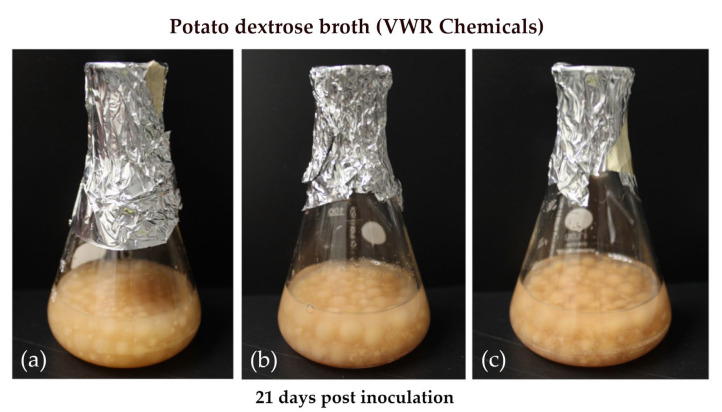
Cultures of *S. chartarum* genotype S strains ATCC 34916 (**a**), IBT 40293 (**b**), and DSM 114129 (**c**) grown in potato dextrose broth.

We also cultured the three strains in broth, a condition that is known to prevent sporulation [34] probably due to the lack of contact with the air [35]. We used potato dextrose broth of VWR Chemicals that, as a solid medium (PDA-V), promoted particularly strong sporulation. Figure 3 shows the well-grown mycelial balls that were obtained after 21 days in broth culture that lacked any detectable sporulation (Table 2).

### 2.2. ATR-IR Measurements

As mentioned above, we have observed halos of different colors in the vicinity of the colonies, but the spore pellets had a similar, black appearance. To further characterize this, we extracted melanin from plugs excised from 21-day-old colonies and recorded infrared spectra (Figure S3 and Table S1). Regardless of the combination of *S. chartarum* isolate and medium, basic bands were observed in all preparations: a strong band at 3300–3260 cm^−1^ corresponding to the primary amine (-NH_2_) or associated –OH, the prominent bands at 2920 and 2851 cm^−1^ representing -CH_3_ and -CH_2_ groups, as expected for organic substances. The band at 1710 cm^−1^ corresponds to an aliphatic ketone (-C=O), and the broader band at 1680 cm^−1^ to 1620 cm^−1^ indicates C=C double bonds. The spectra were very similar to previously described spectra for melanin produced by other filamentous fungi [36,37].

### 2.3. Mycotoxin Cytotoxicity Assessed with an MTT Assay

To examine whether the degree of sporulation is related to cytotoxicity, we tested the extracts from the different agar plates and broth cultures using a swine-kidney cell MTT-test as an unselective cytotoxicity assay. The extracts of the well-sporulated PDA-V three-point cultures were the most cytotoxic samples, independent of the strain tested (Table 3). The extracts of the PDA-V one-point cultures were all less toxic than the extracts of the corresponding three-point cultures. Hence, the plates with three colonies contained more spores and more toxins than those with a solitary colony, which suggests a link between mycotoxin production and sporulation.

Another apparent trend is that the cytotoxicity values of PDA-V cultures were always higher than those of the corresponding PDA-S cultures. The cytotoxicity of PDA-S three-point cultures was even lower than those of the PDA-V one-point cultures, which correlates with the number of spores for IBT 40293 and DSM 114129 (Table S2). The one-point culture of IBT 40293 on PDA-S was more cytotoxic than the other one-point cultures on PDA-S that in turn were only slightly more toxic than those of the broth cultures.

### 2.4. Measurement of Satratoxin G and H Content by HPLC

Since satratoxins are the most cytotoxic metabolites formed by *S. chartarum*, we focused on the two most relevant satratoxins, G and H (SG and SH, respectively), and quantified them by HPLC. The chemical structures of satratoxins G and H are shown in Figure S4.

**Figure 4:**
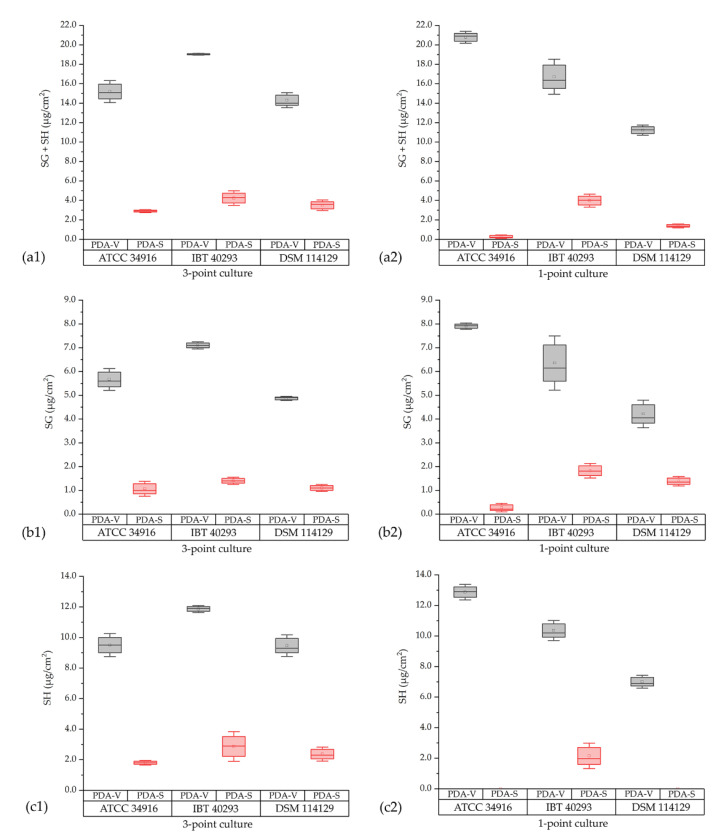
Concentrations of satratoxin G and H measured for cultures of *S. chartarum* genotype S strains ATCC 34916, IBT 40293, and DSM 114129 grown on either PDA-V (gray box plots) or PDA-S (red box plots). The concentrations are normalized to the area of the respective colonies (µg/cm^2^). (**a1**/**a2**): accumulated satratoxin G and H in µg/cm^2^; (**b1**/**b2**): satratoxin G in µg/cm^2^ and (**c1**/**c2**): satratoxin H in µg/cm^2^.

If the amounts of SG and SH are combined (SG + SH (µg/cm^2^)), all strains produced clearly more satratoxins on PDA-V than on PDA-S (Figure 4; gray box plots (PDA-V) vs. red box plots (PDA-S)), irrespective of whether they were grown as three-point or one-point cultures. Strikingly, the one-point cultures on PDA-V (299.4 ± 95.6 µg/agar plate) produced approximately 2.5 times more SG + SH (µg/agar plate) than the three-point cultures on PDA-S (128.9 ± 31.9 µg/agar plate), although the three-point cultures had a colony area that was two times larger (one-point cultures: 18.3 ± 2.1 cm^2^; three-point cultures: 36.0 ± 2.3 cm^2^ (compare Table 1)). The measured concentrations of satratoxins correlate well with the respective spore counts (Figure 2). Hence, we observed the following clear order for the amounts of satratoxins normalized to the colony area: (three-point cultures on PDA-V) > (one-point cultures on PDA-V) > (three-point cultures on PDA-S) > (one-point cultures on PDA-S) > (broth culture in PD-V medium).

In accordance with data from previous studies [27,38,39], the detected concentrations for SH (µg/cm^2^) were generally higher than for SG (µg/cm^2^) (Figure 4). The only exceptions were the one-point cultures of ATCC 34916 and DSM 114129 on PDA-S. In these cases, SG was detected and SH was not detectable. However, a reliable interpretation of these data is difficult since the detected values for SG are in a very low range, above the LOD (signal-to-noise ratio (S/N) ≥ 3) but below the LOQ (S/N ≥ 10).

**Figure 5:**
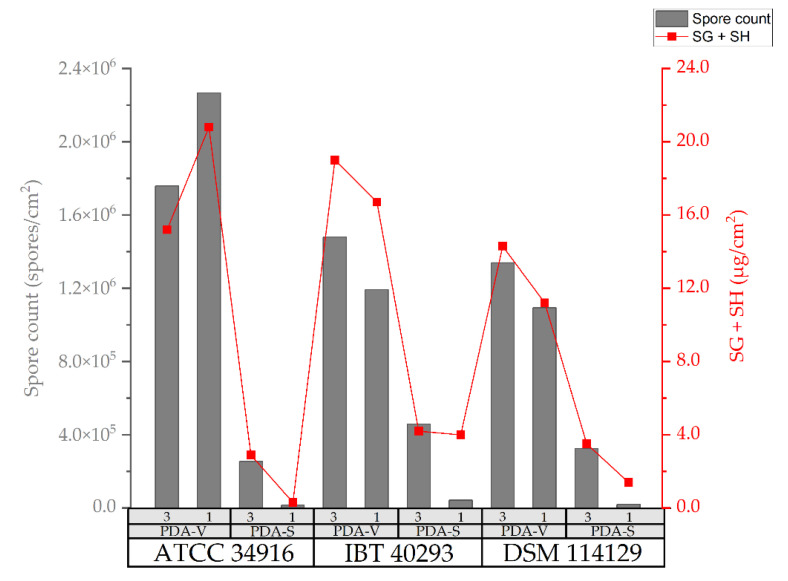
Detected satratoxin concentrations in µg/cm^2^ (red line) of *S. chartarum* genotype S strains (ATCC 34916, IBT 40293, and DSM 114129) on PDA-V and PDA-S three-point cultures (3) and one-point cultures (1) plotted against the respective spore count in spores/cm^2^ (gray columns).

A comparison of the concentrations of the detected satratoxins (µg/cm^2^) and the number of spores (spores/cm^2^) of the different one-point and three-point cultures shows that reduced sporulation correlates with a reduced satratoxin production and increased sporulation correlates with a higher satratoxin concentration. This direct relation between sporulation and satratoxin production is evident from the data presented in Figure 5. We assessed the correlation between the spore count (spores/cm^2^) and the measured concentration of SG + SH (µg/cm^2^) for all strains using the Pearson correlation coefficient. The calculated coefficient for this relationship for all three strains is r ≙ 0.95, which indicates a direct correlation.

### 2.5. Evidence for a Communication between S. chartarum Colonies

**Figure 6:**
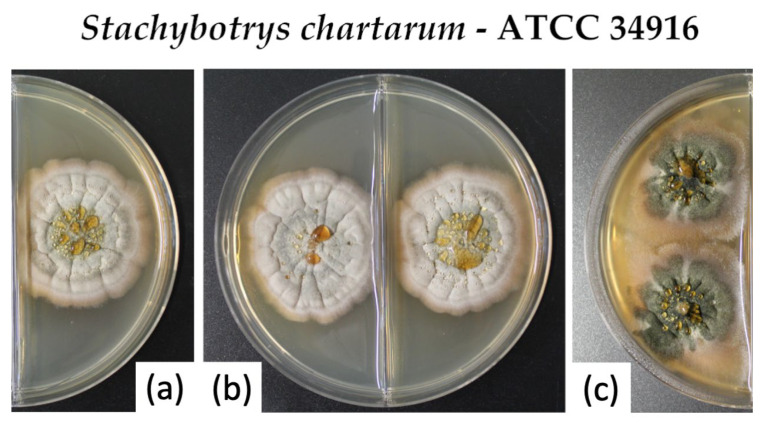
Colonies of the *S. chartarum* genotype S strain ATCC 34916 were grown as one-point culture (**a**) or as a two-point culture that were either neighboring (**c**) or separated by a parting wall (**b**). The cultures were grown on PDA-S.

The results presented above indicate that nutrition media have a significant impact on the sporulation and toxin production of *S. chartarum* but that other factors are also clearly relevant, e.g., the presence of colonies per plate. To gain more information on the communication between colonies, we analyzed whether the relevant signals are exchanged through the air. On this behalf, we separated two colonies of the same strain by a parting wall to restrict communication with volatile substances. The results obtained with strain ATCC 34916 are shown in Figure 6. We found that separated colonies (b) showed only a reduced level of sporulation and thereby resembled an isolated colony (a). The separated colonies showed a clearly reduced sporulation level compared to colonies grown on the same agar (c). These results suggest that the sporulation of neighboring colonies is stimulated either by direct physical contact or by secreted components that are exchanged through the agar.

## 3. Discussion

Secondary metabolites are small molecules produced by microorganisms that are not essential for their growth but supposed to play a role in their internal economy [40]. Mycotoxins are typical secondary metabolites; their production may change depending on different external and internal conditions, such as the availability of nutrients and water [41,42]. For a better understanding of mycotoxins and their biological significance, it is necessary to analyze the mechanisms that trigger or prevent their production. External factors such as temperature and humidity are already known to influence satratoxin production [41,43], but surprisingly little is known about other factors, such as light, pH, and nutrients. This prompted us to launch the current study in order to obtain first insights into the satratoxin production of *S. chartarum*.

The starting point was the finding that two PDA media of different manufacturers led to totally different growth patterns. On PDA-V, the colonies sporulated well and had a black appearance, whereas, on PDA-S, the sporulation of the colonies was sparse.

The only evident difference in composition between the two media used in this study is that PDA-V contains potato peptone as its main component, whereas PDA-S includes potato infusion instead. Both potato peptone and potato infusion are complex mixtures and their ingredients and concentrations are largely unknown. According to Beever and Bollard [44], potato infusion is derived from an aqueous extract of potato tubers. The infusion was described as a suitable source of nitrogen and mineral salts and consequently a good growth factor [44]. Potato peptone is an often-used alternative. With regard to its ingredients, it is better but not well defined. Potato peptone is obtained by hydrolyzation of potato protein. It contains peptides and free amino acids that can serve as a suitable nitrogen source for growth. Nitrogen-rich small molecules are most likely more accessible in potato peptone than in potato infusion. The better growth and sporulation of *S. chartarum* on PDA-V may therefore reflect a better supply of nitrogen in this medium. An influence of nitrogen on the growth and satratoxin production of *S. chartarum* was already suggested by previous data [27,45].

For *Alternaria alternata*, the production of alternariol and other secondary metabolites also depends on the presence of nitrogen and the respective type of nitrogen source [46]. Nitrogen starvation, in turn, was described to induce the production of ochratoxin A in *Aspergillus* spp. and fumonisin in *Fusarium proliferatum*, whereas high amounts of nitrogen impede fumonisin production [47,48,49]. Hence nitrogen has a different impact depending on its concentration, the fungus, and the mycotoxin.

Cultures on PDA-V produced more spores and more satratoxins G and H compared to cultures on PDA-S. The detected satratoxin concentration per cm^2^ of colony area was positively correlated with the number of spores per cm^2^. To provide additional proof for this correlation, we grew the strains in PD broth culture. No sporulation was detectable under this condition. This lack of sporulation has been observed for many fungi. A possible reason that sporulation requires direct contact with the air is that this contact is necessary for an efficient distribution of the conidia.

Both the HPLC data and the cytotoxicity assay results indicate that the satratoxin production in broth was hardly detectable, if not completely absent. Thus, although *S. chartarum* grew well in broth culture, it produced no or only very low amounts of satratoxins. This provides further evidence that sporulation and mycotoxin production are tightly linked in *S. chartarum*. Similar results were previously obtained with *Aspergillus* spp. and their secondary metabolites [30,50,51]. Some mycotoxins of several fungi are known to be associated with sporulation and are secreted by growing colonies [51]. A link between secondary metabolism and sporulation is, therefore, a common feature in many filamentous fungi [30], and both processes may share common regulatory elements [50].

In *Aspergillus* spp., mycotoxin production is regulated by a G-protein signaling pathway [50,52] and similar data were obtained for *Fusarium* species [52]. Intensive genetic research has revealed the signaling circuitry that connects sterigmatocystin-aflatoxin production and sporulation in *A. nidulans*. Both are regulated by two genes, *flu*G and *flb*A, and a loss of function of *flu*G abrogates sporulation and sterigmatocystin production [50]. Whether the same is true for *S. chartarum* is an open question and the lack of genetic tools currently hampers any progress in this field. To date, genetic research on *S. chartarum* largely relies on the analysis of available genomic information, e.g., a comparison of the core trichothecene gene cluster revealed similarities between *Fusarium* and *Stachybotrys* [53]. Since *Fusarium* spp. and *Stachybotrys* spp. produce similar mycotoxin classes, e.g., trichothecenes [25], it is possible that satratoxin production and sporulation may in both species be linked via a G-protein signaling pathway.

In line with previous publications, *S. chartarum* showed a trend to produce more satratoxin H than satratoxin G. The reason for this is unknown, but the pattern was stable and detected using different nutrition media [27,38] or other substrates, such as building materials [39].

A remarkable feature of colonies of *S. chartarum* grown on PDA-S is their green halo. This green halo was also described by Samson and Houbraken [33] using PDA from Difco (BD, Fisher Scientific GmbH) to be a key for discrimination of *S. chartarum* and *S. chlorohalonata*. Our data demonstrate that the appearance of a green halo can also occur with *S*. *chartarum* and is a phenomenon that depends on the type of PDA used (Figure 1 and Figure 2). This result emphasizes that for studies aimed to distinguish *S. chlorohalonata* and *S*. *chartarum,* the PDA type is a critical element.

Our ATR-IR data indicate that *S. chartarum* conidia contain a melanin that was similar in the different spore preparations obtained in this study. This is the first report on the melanin of *S. chartarum*. The black pigment was noted in previous studies, e.g., as a mean that suppresses the ionization during MALDI-TOF MS measurements [34,54], but it was only presumed to be a melanin. The spectra detected in this study are very similar to previously described melanin spectra of other fungi [36,37]. It is, therefore, reasonable to assume that *S. chartarum* produces a typical fungal melanin.

Apart from the correlation between sporulation and satratoxin production, we observed another factor that influences the production of satratoxins. A comparison of one- and three-point cultures revealed that interactions between colonies provide a positive trigger for sporulation and satratoxin production. This is a strong indication that neighboring colonies of the same strain communicate with each other. Such interactions often involve volatile compounds, but our experiments largely exclude this possibility. Our data rather suggest that the communication involves molecules that are secreted into the agar or occur by direct physical contact. The fact that these interactions result in an enhanced mycotoxin production suggests a rather antagonistic character. The colonies seem to be able to perceive a potential competitor for the available resources and respond with the production of satratoxins. These mycotoxins are unlikely to harm another genotype S strain, as in our experimental setting, but they may be able to hamper the growth of other fungi as previously suggested [55]. If these antagonistic responses are indeed triggered by competition for the same resources, the question arises of how hyphae differentiate between hyphae that belong to the same colony and those that belong to a different and rivaling colony. Further experiments are clearly required to investigate these fascinating processes in more detail.

Satratoxin production and sporulation seem to be controlled by a combination of different signals. In general, the effect of the culture medium on sporulation and toxin production was stronger than the impact of inoculation as a three- versus a one-point culture. Other factors, like temperature and humidity, are also involved and contribute to the complicated pattern that determines the production of satratoxins.

Although a previous study showed that spores contain higher concentrations of satratoxin G than mycelia [56], our study is the first to demonstrate a positive correlation between sporulation and satratoxin production for *S. chartarum.* This finding has a direct, practical impact on the diagnostics of indoor mycological samples. If mold contamination is detected or presumed, samples are initially screened for spores and mycelia. A positive result is then followed by an identification of the fungal isolate. For subsequent analytic assays for satratoxin detection, our data strongly recommend using PDA-V or another validated PD medium.

## 4. Conclusions

Culturing *S. chartarum* genotype S on two PDAs from two different manufacturers resulted in different growth patterns, levels of sporulation, and satratoxin production. This has to be considered in future experiments and diagnostic procedures. We provide evidence for a positive correlation between sporulation and satratoxin production. Reduced sporulation correlates with reduced cytotoxicity due to low satratoxin production and vice versa. Due to a lack of genetic tools, this correlation cannot be studied based on the available genetic information but requires other genetic manipulations. The development of such tools is a primary task for the future. Sporulation and satratoxin production are dependent on the nutrition medium and are also influenced by the presence of other cultures on the agar. The enhanced sporulation in the presence of neighboring colonies requires communication and the corresponding signals are apparently exchanged via the aqueous phase and not by volatile compounds. The mechanisms and molecules that are engaged in this interaction are currently unknown and will be the subject of future studies.

## 5. Materials and Methods

### 5.1. Fungal Strains and Culture Conditions

This study analyzed three well-characterized and highly effective satratoxin-producing strains of *S. chartarum* genotype S. They comprise two reference strains (ATCC 34916, IBT 40293) and one field strain (DSM 114129). *S. chartarum* ATCC 34916 was purchased from the American Type Culture Collection (ATCC, Manassas, VA, USA), and *S. chartarum* IBT 40293 was kindly provided by the BioCentrum of the Technical University of Denmark (DTU, Lyngby, Denmark), and *S. chartarum* DSM 114,129 was provided by the Leibniz Institute DSMZ-German Collection of Microorganisms and Cell Cultures (DSMZ, Braunschweig, Germany).

The fungal strains were long-term preserved in sterile 80% glycerol and maintained at −80 °C. Working cultures were grown as three-point cultures on potato dextrose agar (VWR Chemicals, Darmstadt, Germany) for 21 days at 25 °C, a_w_ 0.98 in the dark.

Although the cultures were checked microscopically for their identity, five single spore isolates per strain (≙ five biological replicates) were prepared to ensure that the used strains were pure and to confirm that they were genotype S strains. For this purpose, fungal material from the working cultures was used, and the spores were separated on SNA (Synthetischer Nährstoffarmer Agar; consisting of 0.2 g/L glucose, 0.2 g/L sucrose, 1.0 g/L potassium dihydrogen phosphate, 1.0 g/L potassium nitrate, 0.25 g/L magnesium sulfate anhydrous, 0.5 g/L potassium chloride, 14.0 g/L agar; recipe according to Nirenberg [57]) by spreading them out using an inoculating loop. The SNA plates were incubated for 24 h at 25 °C, a_w_ 0.98 in the dark, until the spores germinated. Then, the germinated spores were localized microscopically, removed using a pipette tip, transferred individually on PDA-V, and grown for 10 d at 25 °C, a_w_ 0.98 in the dark. The genotype of the single spore isolates was confirmed as *S. chartarum* genotype S using the triplex PCR published previously [5]. Afterward, a spore solution derived from every single spore isolate was generated. The spore solutions of one strain were pooled for each strain into one culture and grown for seven days at 25 °C, a_w_ 0.98 in the dark in a cell culture flask. The spore suspensions for each strain were prepared from these cultures by adding 10 mL H_2_O + Tween20 0.01% and 5 glass beads (5 mm, A. Hartenstein GmbH, Würzburg, Germany). The cell culture flasks were shaken vigorously, and the washing solution, including spores, was transferred to a 15 mL flask. Spores were counted using a Neubauer counting chamber, and spore suspensions were diluted to 10^7^ spores/mL.

Subsequently, 10 µL spore suspension was applied by three-point or one-point inoculation on the respective medium, and the cultures were grown for 21 days at 25 °C, a_w_ 0.98 in the dark. The colony areas (cm^2^) were determined for three colonies of three independent agar plates (technical replicates) and the average values were calculated.

The nutrition media used for the three-point and one-point cultures were potato dextrose agar from VWR Chemicals, consisting of 4.0 g/L potato peptone, 20.0 g/L glucose, and 15.0 g/L agar (according to the manufacturer’s ingredient list), and potato dextrose agar from Sigma-Aldrich consisting of 4.0 g/L potato infusion, 20.0 g/L glucose, and 15.0 g/L agar (according to the manufacturer’s ingredient list) (St. Louis, MI, USA). For obvious reasons, harvesting of spores and toxin extraction could not be done from the same agar plate. Thus, we used a second set of plates for the toxin extraction. The data for both agar plate sets are shown in Table S2. Both groups were comparable, and no significant differences were observed between these two plate sets. For the second set of plates, we used a different batch of the media to rule out batch differences.

Liquid cultures were grown in Erlenmeyer flasks by adding 50 mL potato dextrose broth (VWR Chemicals) inoculated with 20 µL of the spore suspension of 10^7^ conidia/mL. Then, the cultures were grown for 21 days in the flask on a laboratory shaker (180 rpm) (LAUDA, GFL Technology, Lauda-Königshofen, Germany). The wet weight was determined during the extraction process.

All media were adjusted to pH 5.6 and sterilized by autoclaving at 121 °C for 20 min before use.

### 5.2. Sample Preparation for Cytotoxicity Assessment and Mycotoxin Analysis

For toxin extraction and purification, each strain was cultured on PDA-V, PDA-S, and in potato dextrose broth, each in triplicate, as described above. Cultures were stored at −20 °C until extraction. For toxin extraction, the content of a whole plate was transferred to a mixing bag (Stomacher^®^ 80 Biomaster Bags, BA6040/STR/DBL strainer double bags, Seward Limited, Worthing, UK). Then, 50 mL ACN/H_2_O (84/16, *v*/*v*) were added (acetonitrile >99.9% and water, HiPerSolv CHROMANORM^®^ for HPLC, VWR International GmbH, Darmstadt, Germany), and bags were treated for 5 min in a bag mixer (Stomacher^®^ 80 micro Biomaster, Seward Limited, Worthing, UK). Subsequently, sample extracts were filtered through a paper filter (Whatman™, 595½, 185 mm in diameter, Maidstone, UK). The wet weight of liquid cultures was determined after the mycelium had been collected from the filter. For cytotoxicity testing, 10 mL of the filtered extracts were evaporated to dryness under a gentle flow of nitrogen at 30 °C (puriVap-6TM, Interchim^®^, Montluçon, France). The samples were then sent from the LMU (Munich, Germany) to the Kazimierz Wielki University (Bydgoszcz, Poland) for testing of the cytotoxicity using a bioassay.

For HPLC measurements, we modified an extraction method described by Hinkley and Jarvis [38]. For this purpose, 15 mL of filtered extracts were mixed with 15 mL hexane (HiPerSolv CHROMANORM^®^ for HPLC, VWR International GmbH, Darmstadt, Germany) and mixed on a laboratory shaker (5 min, 400 rpm) (LAUDA, GFL Technology, Lauda-Königshofen, Germany). Afterward, the extract-hexane mixtures were transferred to separatory funnels (Lenz Laborglas GmbH & Co. KG, Wertheim, Germany) and allowed to stand until three phases (hexane, oily, and extract layer) had completely separated from each other (~30 min). The hexane wash removes oily compounds and improves the purity of the toxin extracts. Then, 10 mL of the toxin extracts (lowest layer) were transferred into reaction tubes and evaporated to dryness under a gentle flow of nitrogen at 30 °C (puriVap-6TM, Interchim^®^, Montluçon, France). The dry extracts were dissolved in 10 mL H_2_O by shaking them on a laboratory shaker (5 min, 400 rpm) followed by ultrasonication (30 min) (Ultrasonic Cleaner USC-T, VWR International GmbH, Darmstadt, Germany). The aqueous toxin extracts were purified using SPE cartridges (Strata™-X 33 µm Polymeric Reversed-Phase 500 mg/6 mL, Phenomenex, Aschaffenburg, Germany) as follows: 1. conditioning: 5 mL methanol (ROTIPURAN^®^ >99.9%, Carl Roth GmbH + Co. KG, Karlsruhe, Germany), 2. equilibration: 5 mL H_2_O (HiPerSolv CHROMANORM^®^ for HPLC, VWR International GmbH, Darmstadt, Germany), 3. loading: 10 mL aqueous extract, 4. washing: 10 mL methanol/H_2_O (30/70, *v*/*v*), 5. elution: 10 mL dichloromethane (LiChrosolv^®^ for liquid chromatography, Merck KGaA, Darmstadt, Germany). The fractions eluted with dichloromethane were evaporated to dryness and dissolved in 1 mL methanol by shaking them on a laboratory shaker (5 min, 400 rpm) with subsequent ultrasonication (30 min). As a final step, the toxin extracts were filtered through a PVDF (polyvinylidene difluoride) syringe filter (0.20 µm, Ø 13 mm, Macherey-Nagel GmbH & Co. KG, Düren, Germany) and transferred to a 1.5-mL glass thread vial with a cap (VWR International GmbH, Darmstadt, Germany).

### 5.3. Sample Preparation for Determination of Spore Count

For spore count determination, three agar plates per strain and nutrition medium (PDA-V or PDA-S) were grown as three-point and one-point cultures (*n* = 36), as described above. Subsequently, 10 mL H_2_O + Tween26 0.01% and 15 glass beads (5 mm, A. Hartenstein GmbH, Würzburg, Germany) were added to each Petri dish. The plates were sealed well with a strip of Parafilm M^®^ (Heathrow Scientific, Vernon Hills, IL, USA) and then shaken (5 min, 350 rpm) on a laboratory shaker (LAUDA, GFL Technology, Lauda-Königshofen, Germany). The washing solution, including spores, was transferred to a 15 mL flask, and the spores were counted using a Neubauer counting chamber.

### 5.4. Melanin Extraction and ATR-Infrared Spectroscopy

For melanin extraction, ATCC 34916, IBT 40293, and DSM 114129 were grown on PDA-V and PDA-S for 21 days at 25 °C, a_w_ 0.98 in the dark. A slightly modified extraction method developed by Gadd was used [58]. Melanin extracts were prepared by cutting a 10 mm diameter plug from 21-day-old colonies and boiling them in 5 mL distilled water for 10 min. The extracts were centrifuged (5000× *g*, 5 min), washed with 5 mL distilled water, and centrifuged again (10,000× *g*, 5 min). The supernatant was discarded, 3 mL 1 M NaOH (Carl Roth GmbH + Co. KG, Karlsruhe, Germany) was added, and the samples were autoclaved (20 min, 121 °C). To precipitate the melanin, the alkaline extract was acidified with concentrated HCl (Carl Roth GmbH + Co. KG, Karlsruhe, Germany) to pH 2. After centrifugation (12,000× *g*, 10 min), the precipitate was washed three times with distilled water and dried under a vacuum at 60 °C (Eppendorf 5301 Concentrator, Hamburg, Germany). The obtained pellets were sent to CVUA Stuttgart (Germany) for attenuated total reflection infrared spectroscopy (ATR-IR) measurements. The samples were placed on the ATR sample zone (base plate diamond) of a Spectrum Two FT-IR Spectrometer (Perkin Elmer, Waltham, MA, USA). Every sample was measured four times in attenuated total reflection mode from 600 to 4000 cm^−1^ with the coupled software (NIOS2). After export, raw data were displayed in Microsoft Excel to obtain the IR spectra (Figure S3).

### 5.5. Cytotoxicity Assessment

Cytotoxicity was measured by the researchers of the Kazimierz Wielki University in Bydgoszcz, Poland, by using the MTT (3-(4,5-dimethylthiazol-2-yl)-2,5-diphenyltetrazolium salt) test. Cells that were not damaged by a mycotoxin can convert a yellow tetrazolium salt, MTT, to a violet, water-insoluble formazan. The reaction takes place in the mitochondria of living cells. The intensity of the color reaction is proportional to the number of intact and metabolically active cells and can be measured photometrically.

The study was performed using a swine-kidney (SK) cell line. The cells were cultured in MEM (Minimum Essential Medium Eagle; Sigma-Aldrich, St. Louis, MI, USA) supplemented with an antibiotic solution (stock solution: 10,000 units of penicillin and 10 mg of streptomycin per mL in 0.9% NaCl (Sigma-Aldrich)), and 5% fetal calf serum (Sigma-Aldrich) in a CO_2_-incubator (CB, BINDER GmbH, Tuttlingen, Germany) (5% CO_2_, 37 °C, 98% humidity). Cells were detached from the bottom of the culture vessel using 0.25% (*w*/*v*) trypsin in 0.53 mM EDTA solution and suspended in the culture medium. Their number was determined using a cell counter (Scepter™ 2.0 Cell Counter, Merck Millipore, Burlington, MA, USA). Subsequently, 2.0 × 10^4^ cells were seeded per well of a 96-well microtiter plate.

Evaporated toxin extracts were dissolved in 1 mL of a mixture of ethanol/dimethyl sulfoxide/MEM (1.7/0.3/98 *v*/*v*/*v*) and different concentrations were prepared using a 2-fold serial dilution method. First, 100 µL of the prepared dilutions were applied to the plate with SK cells and incubated for 48 h at 37 °C in a humidified atmosphere with 5% CO_2_. Then, MTT (3-(4,5-dimethylthiazol-2-yl)-2,5-diphenyltetrazolium bromide) solution (20 µL) was added, and plates were incubated for another 4 h. Subsequently, the supernatant was removed, and 100 μL dimethyl sulfoxide (DMSO) was added to each well. The formation of formazan was measured by spectrophotometric absorbance using an ELISA microplate reader (ELISA LEDETECT 96, Biomed Dr. Wieser GmbH, Salzburg, Austria) at a wavelength of 510 nm (=maximum absorption wavelength of formazan derivatives). The threshold toxicity level was defined as the lowest concentration of the extract that causes a decrease in sample absorption to values <50% of cell metabolic activity.

### 5.6. HPLC Measurement and Method Performance

HPLC analysis of all samples was carried out using a Shimadzu LC-40D linked to a degassing unit (DGU-403), a column oven (CTO-40S), an autosampler (SIL-40C), and a photodiode array detector (SPD-M40) (Shimadzu, Duisburg, Germany). The LabSolutions Lite software (Version 5.99; Shimadzu Deutschland GmbH, Duisburg, Germany) was used for data acquisition and analysis.

Chromatography was performed using a Gemini^®^ C18 110 Å Reversed-Phase LC Column (150 × 4.6 mm, 5 µm, Phenomenex, Aschaffenburg, Germany) attached to a guard column (SecurityGuard™ cartridge, 4 × 3.0 mm internal diameters (ID), Phenomenex, Aschaffenburg, Germany).

The column oven temperature was set to 40 °C, and the maximum operating pressure was 345 bar. The binary gradient consisting of eluent A (water) and eluent B (acetonitrile) (both containing 0.1% formic acid) with a flow rate of 0.4 mL/min and a sample injection volume of 10 µL was applied as follows: 0 min 25% B, 30 min 80% B, 31 min 100% B, 40 min 100% B, 42 min 25% B. The column was equilibrated at starting conditions for 10 min before each run. HPLC-grade water, acetonitrile, and LC-MS-grade formic acid were purchased from VWR International GmbH.

Data collection was performed using a Diode Array Detector. Chromatograms were recorded at 256 nm, and UV/Vis spectra were saved from 200 to 400 nm (Figure S5).

Satratoxin G (SG) and Satratoxin H (SH) standards were purchased from Cayman Chemical (Ann Arbor, MN, USA), and a five-point matrix-matched calibration was performed separately for every nutrition medium tested by preparing five standard matrix solutions (containing 2, 10, 50, 75, or 100 µg/mL). The retention time of SG was 20.8 ± 0.14 min and that of SH was 21.8 ± 0.15 min.

To determine matrix suppression effects, solvent calibration and matrix calibration were done. The matrix effect of PDA-VWR was 1.3% for SG and 6.2% for SH. For PDA-Sigma, it was slightly higher with 3.9% for SG and 8.6% for SH. The highest matrix suppression effect was recorded for potato dextrose broth (12.6% for SG and 21.0% for SH).

The method’s precision was defined by measuring three different concentrations (2, 50, and 100 µg/mL) multiple times (*n* = 15) and comparing the results with the target value. The obtained precision was between 0.4% and 3.8% for SG and 0.3% and 9.2% for SH.

The recovery rate of the extraction method was determined, and the limit of detection (LOD) and limit of quantification (LOQ) were defined. A signal-to-noise ratio (S/N) of ≥3 was determined for LODs and a signal-to-noise ratio (S/N) of ≥10 for the limit of quantification (LOQ). The determined limit of detection for SG was 1.9 µg/agar plate and for SH 10.0 µg/agar plate.

### 5.7. Statistical Analysis

The software OriginPro 2021b (64-bit) SR2 (Version 9.8.5.212) was used for statistical analysis. The correlation between the spore count and the measured concentrations of SG + SH was assessed using the Pearson correlation coefficient.

## Figures and Tables

**Table 1:** Colony size and the amounts of satratoxin G and H measured for three-point and one-point cultures grown on PDA-V and PDA-S. For representative images of these corresponding cultures compare with Figure 1.

	Colony Area	SG	SH	SG + SH	SG	SH	SG + SH
	cm^2^	µg/Agar Plate			µg/cm^2^		
ATCC 34916 PDA-V 3-point culture	51.5 ± 3.6	293.0 ± 6.2	488.4 ± 8.8	781.3 ± 14.9	5.7 ± 0.3	9.5 ± 0.5	15.2 ± 0.8
IBT 40293 PDA-V 3-point culture	48.7 ± 2.4	346.8 ± 11.6	579.0 ± 36.0	925.8 ± 47.6	7.1 ± 0.1	11.9 ± 0.2	19.0 ± 0.1
DSM 114129 PDA-V 3-point culture	46.4 ± 1.7	225.0 ± 6.9	438.4 ± 4.5	663.4 ± 5.9	4.9 ± 0.1	9.5 ± 0.5	14.3 ± 0.5
ATCC 34916 PDA-S 3-point culture	35.9 ± 1.1	38.5 ± 6.7	65.2 ± 2.2	103.7 ± 4.9	1.1 ± 0.2	1.8 ± 0.1	2.9 ± 0.1
IBT 40293 PDA-S 3-point culture	38.3 ± 3.8	53.2 ± 1.1	111.6 ± 33.8	164.8 ± 34.8	1.4 ± 0.1	2.9 ± 0.7	4.2 ± 0.5
DSM 114129 PDA-S 3-point culture	33.7 ± 2.3	37.8 ± 5.9	80.4 ± 11.5	118.3 ± 15.8	1.1 ± 0.1	2.4 ± 0.3	3.5 ± 0.4
ATCC 34916 PDA-V 1-point culture	17.3 ± 0.4	137.2 ± 2.2	223.2 ± 1.3	360.4 ± 1.9	7.9 ± 0.1	12.9 ± 0.3	20.8 ± 0.4
IBT 40293 PDA-V 1-point culture	20.8 ± 0.8	132.7 ± 20.1	215.9 ± 16.5	348.6 ± 36.3	6.4 ± 0.8	10.4 ± 0.4	16.7 ± 1.2
DSM 114129 PDA-V 1-point culture	16.9 ± 0.4	71.2 ± 8.4	118.1 ± 4.5	189.3 ± 10.2	4.2 ± 0.4	7.0 ± 0.3	11.2 ± 0.3
ATCC 34916 PDA-S 1-point culture	9.3 ± 0.0	2.6 ± 1.1 *	n.d.	2.6 ± 1.1	0.3 ± 0.1	n.d.	0.3 ± 0.1
IBT 40293 PDA-S 1-point culture	6.7 ± 0.3	12.2 ± 1.1	14.5 ± 4.5	26.7 ± 4.4	1.8 ± 0.2	2.2 ± 0.6	4.0 ± 0.4
DSM 114129 PDA-S 1-point culture	8.8 ± 0.3	12.2 ± 1.1 *	n.d.	12.2 ± 1.1	1.4 ± 0.1	n.d.	1.4 ± 0.1

SG: satratoxin G, SH: satratoxin H; n.d.: not detectable or rather under the limit of detection (LOD, signal-to-noise ratio (S/N) ≥ 3, LOD for SG: 1.9 µg/agar plate, for SH: 10.0 µg/agar plate); * above the LOD but below the limit of quantification (LOQ, signal-to-noise ratio (S/N) ≥ 10).

**Table 2:** Results obtained for *S. chartarum* genotype S strains ATCC 34916, IBT 40293, and DSM 114129 grown in potato dextrose broth (VWR Chemicals, Darmstadt, Germany).

	Mycelium (Wet Weight)	SG	SH	Spore Count
	mg	µg/Flask		Spores/Flask
ATCC 34916	1203.3 ± 50.3	n.d.	n.d.	n.d.
IBT 40293	1070.0 ± 60.8	n.d.	n.d.	n.d.
DSM 114129	1500.0 ± 52.9	n.d.	n.d.	n.d.

SG: satratoxin G, SH: satratoxin H; n.d.: not detectable or rather under the limit of detection (LOD, signal-to-noise ratio (S/N) ≥ 3, LOD for SG: 1.9 µg/agar plate, for SH: 10.0 µg/agar plate) for the mycotoxins.

**Table 3:** Mycotoxin cytotoxicity assessed with an MTT assay of the extracts obtained from the different cultures on agar plates and broth culture.

Isolate	Culture Medium	Culture Type	Dilution Steps *
IBT 40293	PDA-V	three-point culture	17
		one-point culture	15
	PDA-S	three-point culture	13
		one-point culture	11
	Potato dextrose broth **	fluid	5
ATCC 34916	PDA-V	three-point culture	17
		one-point culture	14
	PDA-S	three-point culture	14
		one-point culture	7
	Potato dextrose broth **	fluid	6
DSM 114129	PDA-V	three-point culture	17
		one-point culture	15
	PDA-S	three-point culture	12
		one-point culture	8
	Potato dextrose broth **	fluid	6

* Dilution steps (2-fold serial dilution method) until the absorbance values decreased below 50% of the controls; PDA-V: potato dextrose agar of VWR Chemicals; PDA-S: potato dextrose agar of Sigma-Aldrich; ** potato dextrose broth of VWR Chemicals.

## Data Availability

Not applicable.

## Supplementary Materials

The following supporting information can be downloaded at: https://www.mdpi.com/article/10.3390/toxins14080515/s1, Figure S1: Microscopy (light microscopy, 200×, lactophenol blue staining) of *S. chartarum* genotype S strain ATCC 34916 as three-point (a and b) and one-point (c and d) culture shows the significantly reduced spore production on PDA-S (b and d) and thinner mycelium compared to the high rate of sporulation with well-grown mycelium on PDA-V (a and c). Figure S2: Spore count per cm^2^ of *S. chartarum* genotype S strains (ATCC 34916, IBT 40293, and DSM 114129) on PDA-V (gray box plots) and PDA-S (red box plots) harvested from the nutrition media shown in Figure 1 (a: three-point cultures, b: one-point cultures). Figure S3: ATR-IR spectra of melanin extracted from *S. chartarum* genotype S strains (ATCC 34916: dark blue and orange, IBT 40293: gray and yellow, DSM 114129: light blue and green) grown on PDA-V and PDA-S (raw data can be found in Table S1). Figure S4: The chemical structures of the macrocyclic trichothecenes satratoxin G (a) and satratoxin H (b) produced by *S. chartarum* genotype S strains (Ulrich 2016). Figure S5: The HPLC chromatograms recorded at 256 nm of satratoxin G (SG) with a retention time (RT) of 20.73 min and satratoxin H (SH) with an RT of 21.62 min diluted in methanol (standard solution) at a concentration of 10.0 µg/mL (1a) with the corresponding UV spectra for SG (1b) and SH (1c) and the detected satratoxin G (15.6 µg/mL, RT: 20.60 min) and satratoxin H (22.9 µg/mL, RT: 21.60 min) in a toxin extract of S. chartarum genotype S strain ATCC 34916 grown on PDA-V as a three-point culture (2a) with the corresponding UV spectra for SG (2b) and SH (2c). Table S1: Raw data of the ATR-IR spectra of melanin extracted from *S. chartarum* genotype S strains (ATCC 34916: dark blue and orange, IBT 40293: gray and yellow, DSM 114129: light blue and green) grown on PDA-V and PDA-S. Table S2: Parameters describing colonies of ATCC 34916, IBT 40293, and DSM 114129 on PDA-V and PDA-S were used to determine the spore count after 21 days of growth (3-point cultures and 1-point cultures).
