# Supplementary material for: Production of Satratoxin G and H Is Tightly Linked to Sporulation in Stachybotrys chartarum
Source: Toxins (Basel). 2022 Jul 28;14(8):515. doi: 10.3390/toxins14080515 (PMC9413001; doi:10.3390/toxins14080515)
Supplement: Supplementary file 1 [file toxins-14-00515-s001.zip › toxins-1834260 supplementary-done.pdf]

toxins

Article

Production of Satratoxin G and H is Tightly Linked to Sporulation in *Stachybotrys chartarum*

Katharina Tribelhorn, Magdalena Twarużek, Ewelina Soszczyńska, Jörg Rau, Christiane Baschien, Reinhard K. Straubinger, Frank Ebel and Sebastian Ulrich

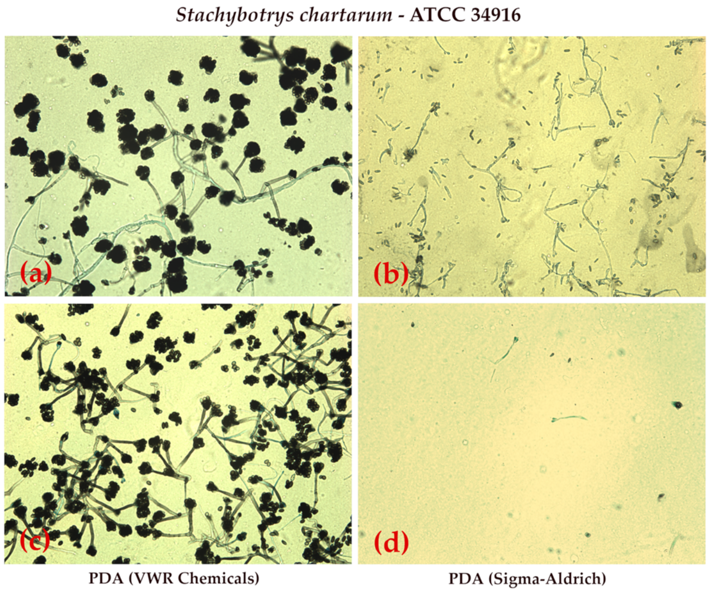

Figure S1. Microscopy (light microscopy, 200x, lactophenol blue staining) of *S. chartarum* genotype S strain ATCC 34916 as three-point (**a and b**) and one-point (**c and d**) culture shows the significantly reduced spore production on PDA-S (b and d) and thinner mycelium compared to the high rate of sporulation with well-grown mycelium on PDA-V (**a and c**).

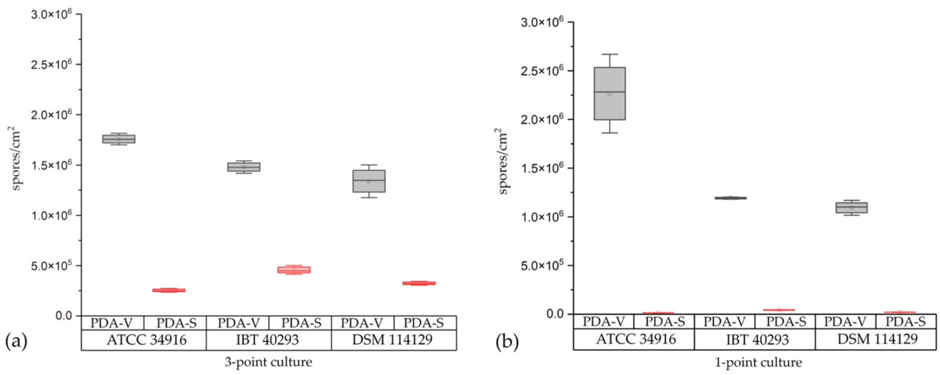

Figure S2. Spore count per cm² of *S. chartarum* genotype S strains (ATCC 34916, IBT 40293, and DSM 114129) on PDA-V (gray box plots) and PDA-S (red box plots) harvested from the nutrition media shown in Figure 1 (**a**: three-point cultures, **b**: one-point cultures).

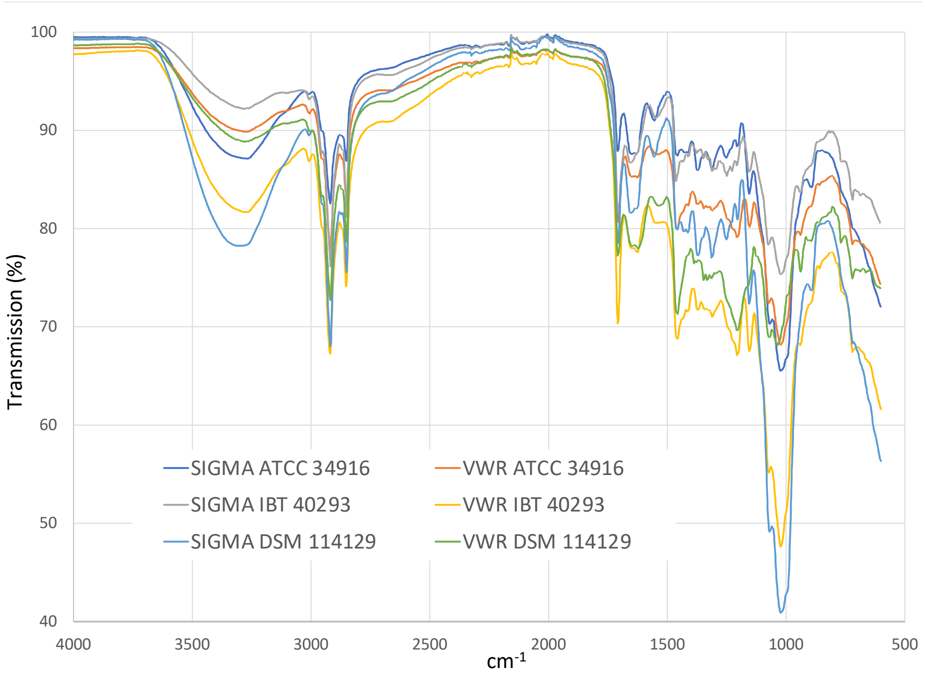

Figure S3. ATR-IR spectra of melanin extracted from *S. chartarum* genotype S strains (ATCC 34916: dark blue and orange, IBT 40293: gray and yellow, DSM 114129: light blue and green) grown on PDA-V and PDA-S (raw data can be found in Table S1).

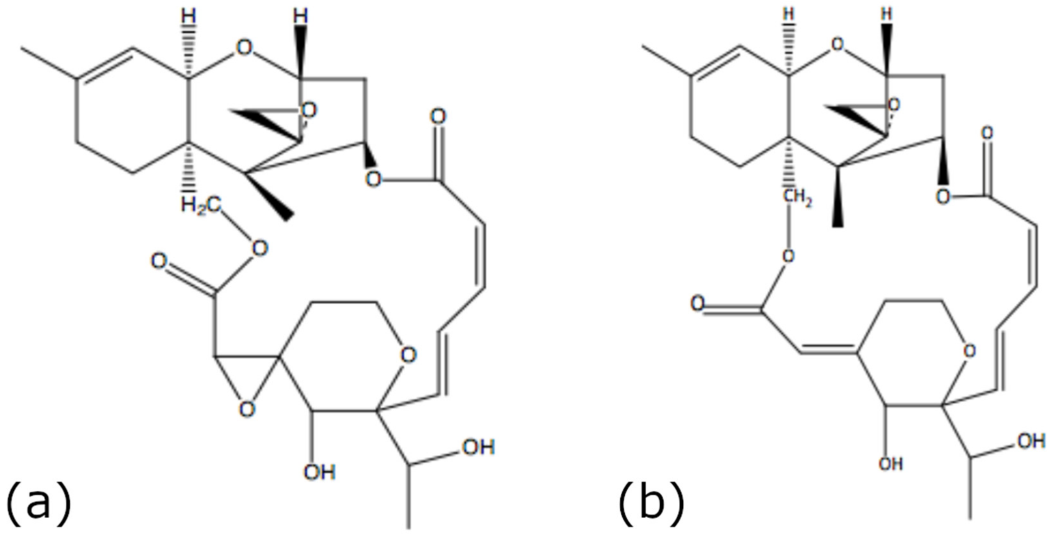

Figure S4. The chemical structures of the macrocyclic trichothecenes satratoxin G (**a**) and satratoxin H (**b**) produced by *S. chartarum* genotype S strains (Ulrich 2016).

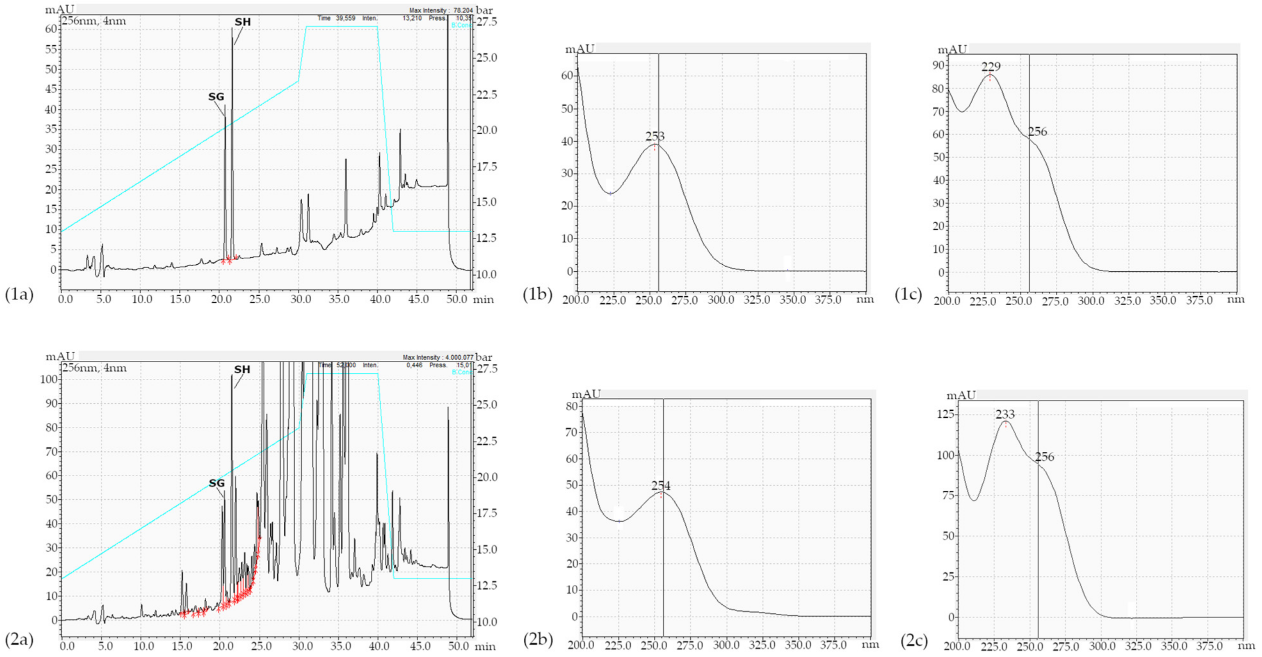

Figure S5. The HPLC chromatograms recorded at 256 nm of satratoxin G (SG) with a retention time (RT) of 20.73 min and satratoxin H (SH) with a RT of 21.62 min diluted in methanol (standard solution) at a concentration of 10.0 µg/mL (**1a)** with the corresponding UV spectra for SG (**1b**) and SH (**1c**) and the detected satratoxin G (15.6 µg/mL, RT: 20.60 min) and satratoxin H (22.9 µg/mL, RT: 21.60 min) in a toxin extract of *S. chartarum* genotype S strain ATCC 34916 grown on PDA-V as a three-point culture (**2a**) with the corresponding UV spectra for SG (**2b**) and SH (**2c**).

Table S2. Parameters describing colonies of ATCC 34916, IBT 40293, and DSM 114129 on PDA-V and PDA-S were used to determine the spore count after 21 days of growth (3-point cultures & 1-point cultures).

	**Colony area**	**Spore count**	
	**cm²**	**spores/Agar Plate**	**spores/cm²**
ATCC 34916<br>PDA-V<br>3-point culture	51.1 ± 0.5	$8.992 \times 10^7 \pm 2.765 \times 10^6$	$1.758 \times 10^6 \pm 3.817 \times 10^4$
IBT 40293<br>PDA-V<br>3-point culture	49.1 ± 2.1	$7.275 \times 10^7 \pm 5.019 \times 10^6$	$1.480 \times 10^6 \pm 4.135 \times 10^4$
DSM 114129<br>PDA-V<br>3-point culture	46.1 ± 0.8	$6.183 \times 10^7 \pm 6.010 \times 10^6$	$1.339 \times 10^6 \pm 1.085 \times 10^5$
ATCC 34916<br>PDA-S<br>3-point culture	36.1 ± 1.1	$9.167 \times 10^6 \pm 2.566 \times 10^5$	$2.544 \times 10^5 \pm 1.244 \times 10^4$
IBT 40293<br>PDA-S<br>3-point culture	38.0 ± 2.7	$1.735 \times 10^7 \pm 1.083 \times 10^6$	$4.572 \times 10^5 \pm 2.892 \times 10^4$
DSM 114129<br>PDA-S<br>3-point culture	34.4 ± 0.2	$1.118 \times 10^7 \pm 4.752 \times 10^5$	$3.248 \times 10^5 \pm 1.278 \times 10^4$
ATCC 34916<br>PDA-V<br>1-point culture	17.3 ± 0.4	$3.933 \times 10^7 \pm 5.150 \times 10^6$	$2.265 \times 10^6 \pm 2.689 \times 10^5$
IBT 40293	20.8 ± 0.4	$2.483 \times 10^7 \pm 6.292 \times 10^5$	$1.192 \times 10^6 \pm 7.351 \times 10^3$

PDA-V<br>1-point culture			
DSM 114129<br>PDA-V<br>1-point culture	16.0 ± 0.2	$1.750 \times 10^7 \pm 1.000$ x10^6	$1.092 \times 10^6 \pm 5.099 \times 10^4$
ATCC 34916<br>PDA-S<br>1-point culture	9.2 ± 0.3	$1.344 \times 10^5 \pm 5.079 \times 10^3$	$1.466 \times 10^4 \pm 6.075 \times 10^2$
IBT 40293<br>PDA-S<br>1-point culture	6.5 ± 0.3	$2.808 \times 10^5 \pm 2.504 \times 10^4$	$4.297 \times 10^4 \pm 1.658 \times 10^3$
DSM 114129<br>PDA-S<br>1-point culture	8.8 ± 0.3	$1.755 \times 10^5 \pm 7.013 \times 10^3$	$1.991 \times 10^4 \pm 6.816 \times 10^2$
